# Interactions between *Paraoxonase 1* Genetic Polymorphisms and Smoking and Their Effects on Oxidative Stress and Lung Cancer Risk in a Korean Population

**DOI:** 10.1371/journal.pone.0119100

**Published:** 2015-03-05

**Authors:** Sang-Yong Eom, Dong-Hyuk Yim, Chul-Ho Lee, Kang-Hyeon Choe, Jin Young An, Kye Young Lee, Yong-Dae Kim, Heon Kim

**Affiliations:** 1 Department of Preventive Medicine and Medical Research Institute, College of Medicine, Chungbuk National University, Cheongju, Korea; 2 Center for Farmers Safety & Health, Chungbuk National University Hospital, Cheongju, Korea; 3 Asbestos Damage Relief Center, Korea Environment Corporation, Incheon, Korea; 4 Department of Internal Medicine and Medical Research Institute, College of Medicine, Chungbuk National University, Cheongju, Korea; 5 Department of Internal Medicine, College of Medicine, Konkuk University, Seoul, Korea; West Virginia University, UNITED STATES

## Abstract

**Background:**

Few studies in epidemiology have evaluated the effects of gene-environment interaction on oxidative stress, even though this interaction is an important etiologic factor in lung carcinogenesis. We investigated the effects of the genetic polymorphisms of paraoxonase 1 (PON1), smoking, and the interaction between the two on lung cancer risk and oxidative stress.

**Methods:**

This study’s subjects consisted of 416 newly diagnosed lung cancer patients and an equal number of matched controls. The GoldenGate assay was used for genotypic analyses of the *PON1* gene. Urinary 8-hydroxydeoxyguanosine (8-OHdG) and thiobarbituric acid reactive substances levels were measured as indicators of oxidative stress.

**Results:**

The *PON1* rs662 AA genotype showed a significantly lower risk of lung cancer than the GG genotype (OR = 0.60, 95% CI: 0.36–0.99). The protective effect of the *PON1* rs662 AA genotype on lung cancer risk was limited to non-smokers. Lung cancer patients who had the rs662 A allele showed a dose-dependent association between smoking status and oxidative stress markers. Among non-smoking lung cancer patients, urinary 8-OHdG levels were significantly lower in individuals with the rs662 GA and AA genotypes than in those with the GG genotype. Furthermore, we found a significant interaction effect between *PON1* rs662 and smoking status on urinary 8-OHdG levels in lung cancer patients.

**Conclusions:**

Our results suggest that the protective effect of *PON1* rs662 SNP against lung carcinogenesis and the induction of oxidative stress might be modulated by the interaction between *PON1* genetic polymorphisms and tobacco smoking.

## Introduction

Of all types of cancer, lung cancer has the highest incidence and mortality worldwide [1]. In Korea, lung cancer is the leading cause of cancer-related death, accounting for approximately one-fourth of all cancer-associated deaths [2]. Tobacco smoking is the most important risk factor for lung cancer, as it is the most likely cause of approximately 90% of lung cancer cases [3,4]. However, only a small proportion of smokers (less than 15%) are diagnosed with lung cancer in their lifetime [3], and approximately 30% of lung cancer patients in Korea are lifelong non-smokers [5]. Thus, although tobacco smoking is a major determinant of lung cancer, it is not sufficient to cause cancer in the absence of additional factors, such as genetic susceptibility and exposure to other carcinogens (i.e., asbestos, nickel, chromium, arsenic, or radon). The carcinogenic effect of smoking is a result of an interaction with additional factors, especially genetic factors [3,4].

Tobacco smoking induces oxidative stress, which can cause severe damage to cellular macromolecules (i.e., DNA, proteins, and lipids) and is a pivotal carcinogenic mechanism linked to various diseases, including lung cancer [6,7]. Oxidative stress is mitigated by exogenous and endogenous antioxidants and antioxidant-defense enzymes (i.e., superoxide dismutase, catalase, glutathione peroxidase, etc.) [8]. Genetic polymorphisms of antioxidant enzymes have an impact on the inter-individual variability in antioxidant defense, which is also associated with susceptibility to various cancers [9].

Paraoxonase 1 (PON1), one of the antioxidant enzymes acting in the blood, plays a key role in preventing the effects of systemic oxidative stress [10–13]. In addition, PON1 is well known for detoxifying the activity of oxons, which are toxic metabolites of organophosphate pesticides [11]. PON1 is predominantly synthesized in the liver and secreted into the bloodstream after binding to high-density lipoproteins (HDL) [11]. The PON1 protein in humans is found in various types of tissue, including lung tissue [14], and PON1 in lung tissue is mainly localized in Clara cells, endothelial cells, and type I cells of the alveolar epithelium [15]. These cells, located in the respiratory portion of the lung, can be exposed to tobacco smoke and reactive oxygen substances released by environmental toxicants [15]. PON1 activity is modulated by various factors, such as genetic polymorphisms, environmental chemicals, pharmaceutical compounds, smoking, alcohol consumption, and dietary factors [12]. It is known that the major determinant of serum PON1 activity is the *PON1* polymorphism [16]. Our previous study showed that approximately 54% of the variance of serum PON1 activity was regulated by the *PON1* Arg192Glu (R192Q, rs662) polymorphism in a Korean population [17].

Recently, a meta-analysis suggested that the *PON1* R192Q polymorphism is a significant risk factor for all cancers, including breast, brain and prostate cancers, especially in Asian populations [18]. At present, only three studies have been conducted to examine the effect of the *PON1* genetic polymorphism on lung cancer risk [19–21]. Although gene-environment interaction could be an important etiologic factor in lung carcinogenesis, there are no epidemiological studies assessing this interaction on oxidative stress.

In this study, we investigated the effects of genetic polymorphisms of *PON1*, smoking, and the interaction between the two on lung carcinogenesis and oxidative stress.

## Materials and Methods

### Study Subjects

The study subjects consisted of 416 newly diagnosed lung cancer patients and an equal number of age- (within 5 years) and sex-matched controls. These patients were histologically confirmed to have lung cancer between January 2001 and October 2008 at the Chungbuk National University Hospital or at the Dankook University Hospital in the Republic of Korea. Control subjects that did not have a previous diagnosis of any type of cancer were selected from individuals receiving routine medical examinations at the two hospitals. After written informed consent was obtained from all subjects, trained interviewers collected information on demographic characteristics, lifestyle factors, and medical and occupational history. Items concerning smoking on the questionnaire included current smoking status, average number of cigarettes smoked daily, total duration of smoking, age of initiating smoking, and age of quitting smoking. Cumulative smoking amount was measured in pack-years (average number of cigarettes smoked daily / 20 × total duration of smoking in years). Non-smokers were defined as individuals who had never smoked cigarettes or who had not smoked more than 100 cigarettes in their lifetime [22]. Peripheral blood and urine were collected from all subjects and then stored at-80°C until the experiment.

### Ethics Statement

This study was approved by the Institutional Review Board of Chungbuk National University Hospital, Republic of Korea (IRB No. 2011–09–072).

### Single nucleotide polymorphisms selection and genotyping analysis

The candidate SNPs (single nucleotide polymorphisms) were selected from 3 public databases: the International HapMap Project database (http://hapmap.ncbi.nlm.nih.gov/), the Functional Element SNPs database (http://sysbio.kribb.re.kr:8080/fesd/index.jsp) [23], and the SNPinfo Web Server (http://snpinfo.niehs.nih.gov/). The selection criteria were as follows: (i) haplotype-tagging SNPs with an R-square cutoff of 0.9 and minimum minor allele frequency in CHB and JPT population of 0.05; (ii) SNPs located in functional regions, such as the promoter, start codon, splice site, coding exon, and stop codon; and (iii) non-synonymous SNPs. Finally, we selected seven SNPs (rs662, rs13306698, rs854572, rs854573, rs854552, rs854565, and rs854568) in the *PON1* gene for genotyping.

Genomic DNA was isolated from peripheral blood using the QuickGene-810 Nucleic Acid Isolation System (Fujifilm, Tokyo, Japan) and the QuickGene DNA Whole Blood Kit in accordance with the manufacturer’s protocol; the DNA samples were stored at-70°C until analysis. SNP genotyping was performed using the VeraCode GoldenGate assay (Illumina, San Diego, CA, USA). All SNPs were in Hardy-Weinberg equilibrium in cases and controls, and the call rate for the seven SNPs was 100%. S1 Table presents detailed information on the seven SNPs and allele frequencies.

### Analysis of biomarkers for oxidative stress

**8-Hydroxydeoxyguanosine**. The level of 8-hydroxydeoxyguanosine (8-OHdG) was measured using an 8-OHdG enzyme-linked immunosorbent assay (ELISA) kit (8-OHdG Check; Japan Institute for the Control of Aging, Fukuroi, Japan). Briefly, urine samples were centrifuged, and 50 μl of the supernatant and 50 μl of an aliquot of the primary antibody were added to an 8-OHdG precoated microplate and incubated at 37°C for 1 hour. The plate was washed 3 times with phosphate-buffered saline. Horseradish peroxidase-conjugated secondary antibody was added to each well, incubated at 37°C for 1 hour, and subsequently washed three times. A 100 μl enzyme substrate containing 3,3′,5,5′-tetra-methyl-benzidine was added, and the plates were incubated at room temperature for 15 minutes under dark conditions. The reaction was terminated by adding of 1 M phosphoric acid, and the absorbance at 450 nm was measured using a microplate reader (GENios; TECAN, Grödig/Salzburg, Austria). The concentration of 8-OHdG was calculated using a standard curve.

**Thiobarbituric acid reactive substances**. Urinary thiobarbituric acid reactive substances (TBARS) were determined using a high-performance liquid chromatographic (HPLC) system with a fluorescence detector [24].21 Briefly, 50 μl of 0.05% butylatedhydroxytoluene, 150 μl of 0.1125 N nitric acid (HNO_3_), and 150 μl of 42 mM thiobarbituric acid were added to a 50μl aliquot of the urine sample or 50 μl of 1,1,3,3-tetramethoxypropane standard and mixed using a vortex. The samples were then heated on a heat block (100°C) for 1 hour and then placed in ice water for 5 minutes to cool; 300 μl of *n*-butanol was subsequently added for the extraction of TBARS, and the samples were then centrifuged at 10,000 × *g* for 5 minutes. Ten microliters of the supernatant was injected into the HPLC system, which consisted of a pump (Lsp 930; Younglin, Seoul, Korea), an automatic injector (SIL 10Avp; Shimadzu, Kyoto, Japan), a fluorescence detector (RF-10AxL; Shimadzu), and a data acquisition module (Autochro-200; Younglin). The columns used were a 150-mm reverse-phase column (TSK-GEL ODS-80TM; Tosoh), and the mobile phases were potassium dihydrogen phosphate:methanol:acetonitrile (60:25:15, v/v/v) at a flow-rate of 1 ml/minute. The excitation/emission wavelengths were 515/553 nm.

### Statistical analysis

Statistical power was calculated using Genetic Power Calculator (http://pngu.mgh.harvard.edu/~purcell/gpc/) [25]. The parameters were set as follows: risk allele frequency of less than 0.15, alpha error of less than 0.05, and a disease prevalence of less than 0.1%. The power of a dominant model was 72.4% when the odds ratio for a genotype with one or two risk allele(s) was taken as 1.5.

Student’s t-test was used to compare continuous variables between the patients and control subjects. Associations between lung cancer and putative risk factors were estimated by odds ratios (ORs) and their corresponding 95% confidence intervals (95% CI) derived from multivariate conditional logistic regression models after adjusting for potential confounding factors, such as age, sex, smoking history, and occupational history. A stratified analysis was used to estimate the combined effects of genotypes and smoking status. The *P*-values for the interactions between the genotypes and smoking status were assessed using the Wald test for the cross-product term in a model containing the main effects of genotype and exposure variable. Multiple testing corrections were carried out using the Benjamini-Hochberg procedure for controlling the false discovery rate (FDR) [26]. All of the statistical analyses were performed using SAS Version 9.2 (SAS Institute, Cary, NC, USA). Linkage disequilibrium statistics and haplotype blocks were obtained using the Haploview program (http://www.broad.mit.edu/mpg/haploview) [27]. Haplotype frequency estimation and the analysis of the association of haplotypes with lung cancer risk were conducted with SNPStats (http://bioinfo.iconcologia.net/SNPStats_web) [28].

## Results

The general characteristics of the 416 lung cancer cases and the 416 age- (within 5 years) and sex-matched controls are presented in Table 1. The lung cancer cases were on average almost 1.8 years older than the controls. The proportions of ex-smokers and current smokers were significantly higher in the lung cancer cases than in the controls, and smoking status was significantly associated with an increased risk of lung cancer. The mean of the cumulative smoking amount in lung cancer cases was about twice as high than that of controls, and cumulative smoking amount significantly increase the risk of lung cancer in a dose-dependent manner. The geometric means for urinary TBARS and 8-OHdG were not significantly different between lung cancer cases and controls.

**Table 1:** General characteristics of lung cancer cases and controls.

Variables	Cases	Controls	*P*-value or OR (95% CI)
Age, years, mean ± SD	66.2 ± 9.8	64.4 ± 9.9	0.011
Gender, N (%)			1.000
Male	324 (77.9)	324 (77.9)	
Female	92 (22.1)	92 (22.1)	
Body mass index, kg/m2, mean ± SD	22.3 ± 3.4	23.7 ± 4.3	<0.001
Smoking status, N (%)			
Non-smokers	72 (17.3)	157 (37.7)	1.00 (ref.)
Ex-smokers	185 (44.5)	142 (34.1)	5.14 (3.19, 8.28)^a^
Current smokers	159 (38.2)	117 (28.1)	5.60 (3.45, 9.09) ^a^
Cumulative smoking amounts, pack-years, mean ± SD	39.1 ± 29.1	19.9 ± 23.6	<0.001
Cumulative smoking amounts, N (%)			
0 pack-years (Non-smokers)	72 (17.3)	157 (38.1)	1.00 (ref.)
<30 pack-years	81 (19.5)	127 (30.8)	2.84 (1.73, 4.68) ^a^
≥30 pack-years	263 (63.2)	128 (31.1)	11.34 (6.80, 18.90) ^a^
Alcohol consumption, N (%)			
Non-drinker	185 (44.6)	167 (40.6)	1.00 (ref.)
Drinker	230 (55.4)	244 (59.4)	0.84 (0.62, 1.14)^b^
8-OHdG, μg/g creatinine, GM (95% CI)	5.06 (4.55, 5.62)	4.81 (4.43, 5.22)	0.457
TBARS, μmol/g creatinine, GM (95% CI)	0.88 (0.79, 0.98)	0.84 (0.77, 0.92)	0.486

TBARS: thiobarbituric acid reactive substances; 8-OHdG: 8-hydroxydeoxyguanosine; GM: geometric mean; CI: confidence intervals.

^a^Adjusted for age and sex.

^b^Adjusted for age, sex and smoking status.

^c^Individuals who have work experience in occupations related lung cancer risk, such as petrochemicals, construction, mining, asbestos or rockwool production, welding, electrical manufacture, plastic or rubber manufacture, smelting, and asphalt.

^d^Reference category is all other occupations.

The distributions of the seven SNPs (rs662, rs13306698, rs854572, rs854573, rs854552, rs854565, and rs854568) in the *PON1* gene among lung cancer cases and controls are shown in Table 2. The rs662 AA (192QQ) genotype showed a significantly lower risk for lung cancer than the GG (192RR) genotype (OR = 0.60, 95% CI: 0.36–0.99). The rs854565 GA genotype also showed a marginal association with reduced lung cancer risk when compared with GG genotype (OR = 0.77, 95% CI: 0.54–1.04). However, neither of those associations were statistically significant after controlling for the FDR.

**Table 2:** Associations between seven genetic polymorphisms of *PON1* and the risk of lung cancer.

SNP ID	Genotypes	Cases, N (%)	Controls, N (%)	OR^a^ (95% CI)	P-value	FDR
rs13306698	AA	354 (85.1)	357 (85.8)	1.00 (ref.)		
	AG	60 (14.4)	58 (13.9)	1.02 (0.68, 1.53)	0.944	0.944
	GG	2 (0.5)	1 (0.2)	2.07 (0.19, 23.02)	0.553	0.863
	AG+GG	62 (14.9)	59 (14.2)	1.03 (0.69, 1.55)	0.872	0.872
rs662	GG	209 (50.2)	180 (43.3)	1.00 (ref.)		
	GA	170 (40.9)	188 (45.2)	0.77 (0.57, 1.04)	0.087	0.312
	AA	37 (8.9)	48 (11.5)	0.60 (0.36, 0.99)	0.044	0.308
	GA+AA	207 (49.8)	236 (56.7)	0.73 (0.55, 0.98)	0.034	0.238
rs854552	TT	234 (56.3)	237 (57)	1.00 (ref.)		
	TC	147 (35.3)	149 (35.8)	1.04 (0.76, 1.41)	0.808	0.943
	CC	35 (8.4)	30 (7.2)	1.14 (0.66, 1.97)	0.637	0.863
	TC+CC	182 (43.8)	179 (43.0)	1.06 (0.79, 1.41)	0.709	0.872
rs854565	GG	236 (56.7)	215 (51.7)	1.00 (ref.)		
	GA	152 (36.5)	172 (41.4)	0.77 (0.57, 1.04)	0.089	0.312
	AA	28 (6.7)	29 (7.0)	0.82 (0.45, 1.47)	0.500	0.863
	GA+AA	180 (43.3)	201 (48.3)	0.78 (0.58, 1.04)	0.086	0.301
rs854568	AA	190 (45.7)	198 (47.6)	1.00 (ref.)		
	AG	182 (43.8)	178 (42.8)	1.10 (0.81, 1.48)	0.553	0.774
	GG	44 (10.6)	40 (9.6)	1.03 (0.63, 1.69)	0.903	0.903
	AG+GG	226 (54.3)	218 (52.4)	1.08 (0.81, 1.44)	0.586	0.820
rs854572	CC	103 (24.8)	124 (29.8)	1.00 (ref.)		
	CG	208 (50.0)	201 (48.3)	1.23 (0.87, 1.73)	0.244	0.569
	GG	105 (25.2)	91 (21.9)	1.36 (0.91, 2.03)	0.139	0.487
	CG+GG	313 (75.2)	292 (70.2)	1.27 (0.92, 1.75)	0.150	0.350
rs854573	TT	329 (79.1)	322 (77.4)	1.00 (ref.)		
	TC	82 (19.7)	89 (21.4)	0.89 (0.62, 1.27)	0.519	0.774
	CC	5 (1.2)	5 (1.2)	0.80 (0.22, 2.92)	0.740	0.863
	TC+CC	87 (20.9)	94 (22.6)	0.89 (0.63, 1.25)	0.488	0.820

^a^Adjusted for age, sex, smoking status, and occupational history.

^b^False discovery rate adjusted using the Benjamini-Hochberg procedure[26].

Table 3 shows the effect of *PON1* SNPs on lung cancer risk according to smoking status. In non-smokers, the rs662 AA (192QQ) genotype exhibited a significantly reduced lung cancer risk (OR = 0.25, 95% CI: 0.06–0.98), and the lung cancer risk significantly decreased as the number of rs662 A alleles increased (p = 0.047). In current and ex-smokers, however, no statistically significant association was found. When stratified according to smoking status, the rs13306698, rs854552, rs854565 and rs854568 genotypes were not associated with lung cancer risk for any smoking status. In addition, no interaction between *PON1* SNPs and smoking status was significantly associated with lung cancer risk.

**Table 3:** Associations between seven genetic polymorphisms of *PON1* and the risk of lung cancer, according to smoking status.

SNP ID	Genotypes	Non-smokers		Current or ex-smokers		*P*_for interaction_
		Cases/controls	OR (95% CI)	Cases/controls	OR (95% CI)	
rs13306698	AA	60/138	1.00 (ref.)	294/219	1.00 (ref.)	0.451
	AG	11/19	1.44 (0.59, 3.50)	49/39	0.95 (0.60, 1.51)	
	GG	1/0	-	1/1	1.04 (0.07, 16.84)	
	AG+GG	12/19	1.56 (0.65, 3.72)	50/40	0.95 (0.60, 1.51)	
P _trend_ ^b^			0.241		0.839	
rs662	GG (RR)	39/69	1.00 (ref.)	170/111	1.00 (ref.)	0.454
	GA (QR)	30/70	0.73 (0.39, 1.37)	140/118	0.78 (0.55, 1.10)	
	AA (QQ)	3/18	0.25 (0.06, 0.98)	34/30	0.72 (0.41, 1.26)	
	GA+AA	33/88	0.63 (0.34, 1.15)	174/148	0.77 (0.55, 1.07)	
P _trend_ ^b^			0.047		0.125	
rs854552	TT	35/89	1.00 (ref.)	199/148	1.00 (ref.)	0.253
	TC	30/59	1.49 (0.78, 2.86)	117/90	0.98 (0.68, 1.39)	
	CC	7/9	2.11 (0.65, 6.81)	28/21	1.03 (0.56, 1.91)	
	TC+CC	37/68	1.58 (0.85, 2.93)	145/111	0.99 (0.71, 1.37)	
P _trend_ ^b^			0.122		0.986	
rs854565	GG	45/87	1.00 (ref.)	191/128	1.00 (ref.)	0.443
	GA	26/57	0.88 (0.46, 1.67)	126/115	0.74 (0.52, 1.04)	
	AA	1/13	0.12 (0.01, 1.09)	27/16	1.15 (0.59, 2.24)	
	GA+AA	27/70	0.74 (0.40, 1.38)	153/131	0.79 (0.57, 1.10)	
P _trend_ ^b^			0.117		0.430	
rs854568	AA	27/77	1.00 (ref.)	163/121	1.00 (ref.)	0.390
	AG	39/68	1.51 (0.80, 2.85)	143/110	0.98 (0.69, 1.39)	
	GG	6/12	0.95 (0.30, 3.00)	38/28	1.00 (0.58, 1.74)	
	AG+GG	45/80	1.41 (0.76, 2.61)	181/138	0.99 (0.71, 1.37)	
P _trend_ ^b^			0.523		0.968	
rs854572	CC	16/46	1.00 (ref.)	87/78	1.00 (ref.)	0.857
	CG	39/78	1.35 (0.64, 2.83)	169/123	1.16 (0.79, 1.72)	
	GG	17/33	1.49 (0.61, 3.63)	88/58	1.30 (0.82, 2.06)	
	CG+GG	56/111	1.39 (0.69, 2.81)	257/181	1.21 (0.84, 1.74)	
P _trend_			0.366		0.262	
rs854573	TT	60/123	1.00 (ref.)	269/199	1.00 (ref.)	0.392
	TC	12/32	0.72 (0.32, 1.61)	70/57	0.90 (0.60, 1.35)	
	CC	0/2	-	5/3	1.15 (0.27, 4.91)	
	TC+CC	12/34	0.64 (0.29, 1.43)	75/60	0.92 (0.62, 1.36)	
P _trend_			0.195		0.733	

^a^Adjusted for age, sex, smoking status, and occupational history.

^b^For the linear trend across all three genotypes.

The rs662, rs13306698, and rs854565 genotypes showed a strong LD to one another (D’ >0.9). The strongest LD was found in two non-synonymous SNPs, rs13306698 and rs662 (D’ = 0.998), which were then selected for haplotype analysis (S1 Fig.). After adjusting for age, sex, smoking status, and occupational history, the A-A haplotype was significantly associated with lung cancer risk compared with the A-G haplotype in the additive genetic model (OR = 0.77, 95% CI: 0.61–0.96). However, after stratifying by smoking status, there were no haplotypes associated with the risk of lung cancer (Table 4).

**Table 4:** Associations between the haplotypes of two non-synonymous SNPs of *PON1* and the risk of lung cancer, according to smoking status

Haplotypes (rs1330669, rs662)	All subjects		Non-smokers		Current or ex-smokers		*P*_for interaction_
	Frequency of cases/controls	OR (95% CI)^a^	Frequency of cases/controls	OR (95% CI)^b^	Frequency of cases/controls	OR (95% CI) ^b^	
A-G	0.59/0.61	1.00 (ref.)	0.66/0.60	1.00 (ref.)	0.62/0.58	1.00 (ref.)	0.570
A-A	0.34/0.32	0.77 (0.61–0.96)	0.25/0.34	0.63 (0.38–1.05)	0.30/0.34	0.81 (0.63–1.05)	
G-G	0.07/0.07	0.95 (0.64–1.42)	0.09/0.06	1.35 (0.57–3.18)	0.07/0.08	0.89 (0.57–1.40)	

^a^Adjusted for age, sex, smoking status, and occupational history.

^b^Adjusted for age, sex, and occupational history.

**Fig 1:**
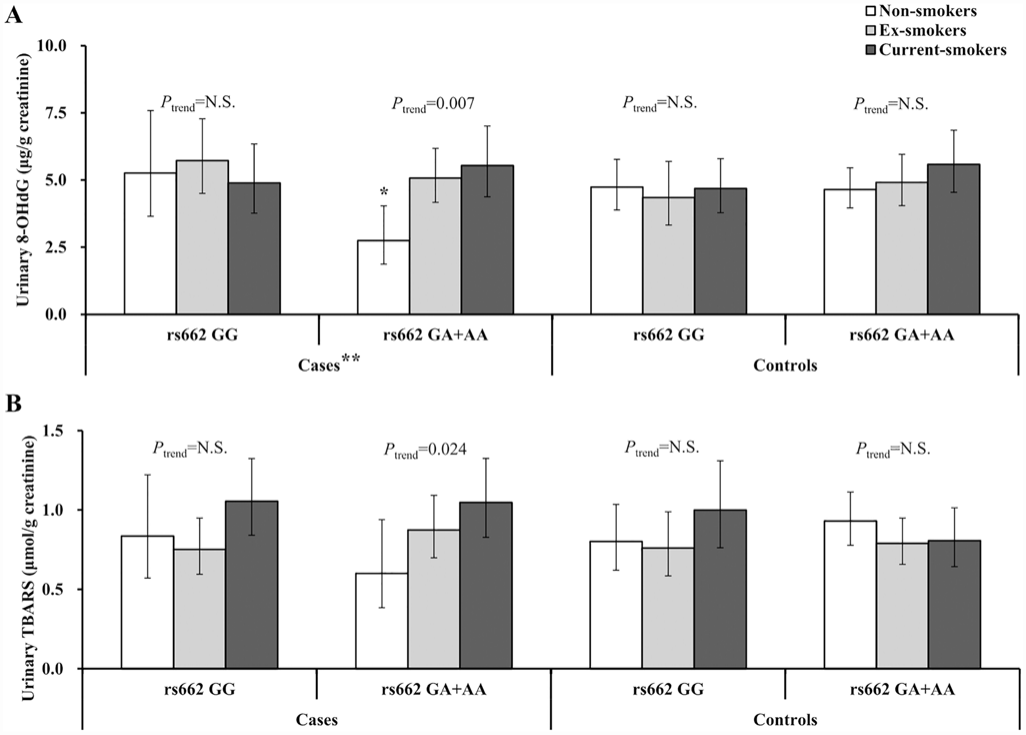
Geometric means and 95% confidence intervals for urinary oxidative stress markers (A: 8-hydroxydeoxyguanosine, B: thiobarbituric acid reactive substances) according to smoking status and *PON1* rs662 SNP in lung cancer patients and controls. *Significant difference between the genotypes in non-smokers (P = 0.017), ** Significant interaction (SNP×smoking)(P = 0.025).

Fig. 1 presents the levels of oxidative stress biomarkers in the lung cancer patients and controls, according to *PON1* rs662 SNP and smoking status. In lung cancer patients with one or two *PON1* rs662 A (192Q) alleles, 8-OHdG and TBARS levels showed a significant positive exposure-response trend for smoking status (*P*_*trend*_ = 0.007 and 0.024, respectively), but this trend was not found in lung cancer patients with the *PON1* rs662 GG (192RR) genotype and controls. Among non-smoker patients, urinary 8-OHdG level was significantly lower in individuals with the rs662 GA (192RQ) and AA (192QQ) genotypes than in those with the GG (192RR) genotype. We found a significant interaction effect between *PON1* rs662 SNP and smoking status on the urinary 8-OHdG level in lung cancer patients (*P*_*interaction*_ = 0.025). These significant interactions were not identified for *PON1* rs854572, rs854573, rs854552, rs854565, or rs854568 SNPs (data not shown).

In addition, we tested the influence of the *PON1* SNPs on lung cancer risk after stratification according to histological type. The odds ratio of the rs662 GA + AA (192RQ + QQ) genotype in the adenocarcinoma group (OR = 0.59, p = 0.053) was marginally significant and lower than for other histological types (squamous cell carcinoma OR = 0.76, p = 0.246; other non-small cell carcinoma OR = 0.90, p = 0.658) (data not shown).

## Discussion

This case-control study found that the *PON1* rs662 (R192Q) polymorphism is associated with a risk of lung cancer, especially among non-smokers. Furthermore, we observed a significant interaction between the *PON1* rs662 SNP and smoking on oxidative stress in lung cancer patients.

The results of this study indicate that the individuals with one or two rs662 A (192Q) alleles of the *PON1* rs662 polymorphism showed a reduced risk for lung cancer. Until now, several epidemiological studies have reported that the *PON1* genetic polymorphism is associated with lung cancer risk [19–21] or reduction of lung function [29]. Among them, the two most recent studies suggested that the 192Q allele of the *PON1* is a potential protective factor against lung cancer [20,21], which is in concordance with this present study. Similarly, the *PON1* 192Q allele has been reported to reduce the risk of bladder cancer [30], ovarian cancer [31], and B-cell lymphoma [32]; in contrast, it has been reported to increase the risk of lung [19], breast [33] and prostate cancers [34].

Cigarette smoking decreases serum PON1 activity in humans [12,17], and cigarette smoke extracts inhibit serum PON1 activity through the modification of the free thiol residue at amino acid 283 in PON [35]. In addition, a recent study reported that myeloperoxidase (MPO), a heme enzyme abundantly produced by neutrophils, inactivates PON1 functioning [36]. The number of neutrophils was found to be higher in current or ex-smokers than in non-smokers [37], with greater MPO activity in smokers than in non-smokers [38]. These results indicate that the serum PON1 activity of individuals exposed to tobacco smoke would be inhibited regardless of their *PON1* genotype. This supports our data which indicate that the *PON1* rs662 polymorphism was significantly associated with lung cancer risk in non-smokers, but not in ex- or current smokers. Furthermore, a similar association was also observed between the *PON1* rs662 polymorphism and oxidative stress level in lung cancer patients. The *PON1* rs662 A (192Q) allele was associated with a significant reduction in the urinary 8-OHdG level of non-smoking lung cancer patients, but this protective allelic effect was not observed in current or ex-smokers with lung cancer.

The effect of the *PON1* rs662 polymorphism on the risk of lung cancer differed according to the smoking status. Particularly, non-smokers with *PON1* rs662 AA (192QQ) genotype had a significant 75% decrease in lung cancer risk (OR = 0.25, 95% CI [0.06–0.98]), but this reduction was not observed in current or ex-smokers (OR = 0.72, 95% CI [0.41–1.26]). However, the interaction between the *PON1* rs662 polymorphism and smoking habits on lung cancer risk was not statistically significant, probably owing to the relatively small sample size. Nevertheless, our results showed a clear difference in the magnitude of lung cancer risk across *PON1* rs662 genotypes according to the smoking status. This is suggestive of a possible gene-environment interaction that influences lung cancer risk.

The protective effect of the *PON1* rs662 SNP on 8-OHdG which could be modified by smoking habits was statistically significant only in lung cancer patients, but not in controls. This finding suggests the possibility that the *PON1* 192Q allele reduces the lung cancer risk of non-smoking individuals by protecting against oxidative stress [7]. However, it is not certain that the protective effect of the *PON1* rs662 SNP against oxidative stress exists in the body of lung cancer patients before the start of carcinogenesis. We cannot rule out the possibility that lung cancer can change the action of the *PON1* 192Q enzyme on oxidative stress. These results, therefore, need to be interpreted with caution and should be further investigated with prospective studies.

PON1 is an antioxidant enzyme that can act as a scavenger for systemic oxidative stress [10–13]. Previous studies have reported associations between *PON1* genetic polymorphisms or PON1 enzyme activity and oxidative stress, but the results are still inconclusive. Serum PON1 activity has been negatively correlated with urinary 8-OHdG levels in patients with Alzheimer’s disease [39] and laryngeal squamous cell carcinoma [40]. Bhattacharyya et al. found that the *PON1* 192 RR genotype was associated with a lower level of oxidative stress [13], and Ji et al. similarly reported that carriers of the *PON1* rs662 Q allele have a significantly higher level of 8-OHdG in sperm DNA than R allele carriers [41]. In contrast, Min et al. reported that individuals carrying the *PON1* 192RR genotypes showed a higher level of urinary 8-OHdG than those with other genotypes [42]. Our study found that non-smoker lung cancer patients with the *PON1* 192RR genotype showed a significantly higher level of urinary 8-OHdG than those with the 192RQ and QQ genotypes.

The *PON1* rs662 (R192Q) polymorphism significantly modifies the catalytic efficiency of PON1 in a substrate-dependent manner [12]. The *PON1* 192R allele hydrolyzes paraoxon and chlorpyrifos oxon more efficiently than the *PON1* 192Q allele *in vitro*, while diazoxon, sarin, and soman are hydrolyzed more rapidly by the *PON1* 192Q allele than the *PON1* 192R allele [17,43]. The PON1 192Q-alloenzyme protects low-density lipoproteins from oxidative modification more effectively when compared to the R-alloenzyme [44], and arylesterase activity of PON1 is associated with antioxidant capacity to a greater degree than with paraoxonase activity [45,46]. There is substantial evidence that the *PON1* R192Q polymorphism plays an important role in enzyme activity; specifically, this genetic polymorphism contributes to HDL binding and stability of PON1 [47]. In this study, the *PON1* 192Q allele was associated with lower oxidative stress in non-smoking lung cancer patients, and with an overall reduction in lung cancer risk. Our previous study showed that PON1-paraoxonase activity in Koreans with the *PON1* 192RR genotype was higher than in those with the QQ genotype, and that PON1-arylesterase activity in those with the *PON1* 192QQ genotype was higher than in those with the 192RR genotype [17]. Therefore, our present findings suggest that the *PON1* 192Q allele might reduce lung cancer risk or oxidative stress through increased PON1-arylesterase activity.

Previous studies reported that distributions of SNPs and serum PON1 activity were not different between histological types of lung cancer [21,48]. However, in our stratified analyses according to the histological types, differing associations were observed between *PON1* rs662 SNP and lung cancer risk for different histological types, although statistical significance was not reached, probably owing to the small sample size. *PON1* rs662 SNP was marginally associated with lung cancer risk solely in the adenocarcinoma group, which was more common in non-smokers. Concordantly, our findings showed that the *PON1* rs662 SNP was associated with a significant reduction in the lung cancer risk of non-smokers. These facts suggest that *PON1* rs662 SNP may be considered as a genetic susceptibility marker for lung cancer in non-smokers.

In conclusion, the results of this study suggest that functional polymorphisms of *PON1* may be associated with the risk of lung cancer and that the effect of *PON1* polymorphisms on lung carcinogenesis and oxidative stress may be modulated by tobacco smoking.

## Supporting Information

S1 Fig Linkage disequilibrium plot and D’ statistics of the seven *PON1* SNPs. (DOCX)

S1 Table Information about the 7 SNPs and allele frequencies selected in this study. (DOCX)
