# Supplementary figures and images for: Interactions between Paraoxonase 1 Genetic Polymorphisms and Smoking and Their Effects on Oxidative Stress and Lung Cancer Risk in a Korean Population
Source: PLoS One. 2015 Mar 5;10(3):e0119100. doi: 10.1371/journal.pone.0119100 (PMC4350985; doi:10.1371/journal.pone.0119100)

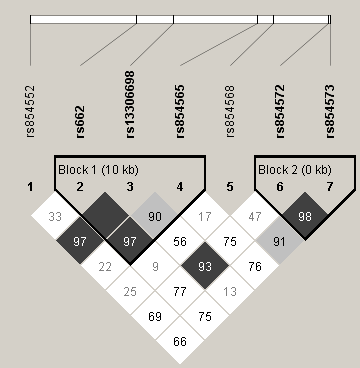


**S1 Fig**. Linkage disequilibrium plot and D’ statistics of the seven *PON1* SNPs

Supplement: S1 Fig — (DOCX) [file pone.0119100.s001.docx]
