# Supplementary material for: Interactions between Paraoxonase 1 Genetic Polymorphisms and Smoking and Their Effects on Oxidative Stress and Lung Cancer Risk in a Korean Population
Source: PLoS One. 2015 Mar 5;10(3):e0119100. doi: 10.1371/journal.pone.0119100 (PMC4350985; doi:10.1371/journal.pone.0119100)
Supplement: S1 Table — (DOCX) [file pone.0119100.s002.docx]

**S1 Table.** Information about the 7 SNPs and allele frequencies selected in this study

| SNPID | Gene Symbol | Position in Chr. 7 | Location | Amino acid change | Tag SNP^a^ | Allele | | Cases |  | Controls |  |
| --- | --- | --- | --- | --- | --- | --- | --- | --- | --- | --- | --- |
|  |  |  |  |  |  | Major | Minor | MAF | *p* for HWE | MAF | *p* for HWE |
| rs13306698 | *PON1* | 94940782(-) | Exon 5 | R160G | Yes | A (R) | G (G) | 0.08 | 0.750 | 0.07 | 0.394 |
| rs662 | *PON1* | 94937446(-) | Exon 6 | R192Q | Yes | G (R) | A (Q) | 0.29 | 0.773 | 0.34 | 0.918 |
| rs854552 | *PON1* | 94927924(+) | UTR | - | Yes | T | C | 0.26 | 0.088 | 0.25 | 0.328 |
| rs854565 | *PON1* | 89555380(+) | Intron | - | Yes | G | A | 0.25 | 0.601 | 0.28 | 0.494 |
| rs854568 | *PON1* | 89556841(+) | Intron | - | Yes | A | G | 0.32 | 0.966 | 0.31 | 1.000 |
| rs854572 | *PON1* | 89561734(+) | Intergenic | - | No | C | G | 0.50 | 1.000 | 0.46 | 0.574 |
| rs854573 | *PON1* | 89561901(+) | Intergenic | - | No | T | C | 0.11 | 0.966 | 0.12 | 0.677 |

MAF: Minor allele frequency, HWE: Hardy-Weinberg equilibrium, R: Arginine, G: Glycine, Q: Glutamine, UTR: untranslated region

^a^Based on the International HapMap Project database (<http://hapmap.ncbi.nlm.nih.gov/>, HapMap Data Rel 27, Population: CHB and JPT, R^2^>0.9, MAF>0.05
